# Targeted Knockout of Eukaryotic Translation Initiation Factor 4E Confers Bymovirus Resistance in Winter Barley

**DOI:** 10.3389/fgeed.2021.784233

**Published:** 2021-11-29

**Authors:** Robert Eric Hoffie, Ingrid Otto, Dragan Perovic, Nagaveni Budhagatapalli, Antje Habekuß, Frank Ordon, Jochen Kumlehn

**Affiliations:** ^1^ Plant Reproductive Biology, Leibniz-Institute of Plant Genetics and Crop Plant Research (IPK), Gatersleben, Germany; ^2^ Institute for Resistance Research and Stress Tolerance, Julius Kuehn Institute (JKI), Federal Research Centre for Cultivated Plants, Quedlinburg, Germany

**Keywords:** Cas9, CRISPR, genome editing, RNA-guided endonucleases, targeted mutagenesis, protoplasts, doubled haploid

## Abstract

The Eukaryotic Translation Initiation Factor 4E (EIF4E) is a well-known susceptibility factor for potyvirus infections in many plant species. The barley yellow mosaic virus disease, caused by the bymoviruses *Barley yellow mosaic virus* (BaYMV) and *Barley mild mosaic virus* (BaMMV), can lead to yield losses of up to 50% in winter barley. In autumn, the roots of young barley plants are infected by the soil-borne plasmodiophoraceous parasite *Polymyxa graminis* L. that serves as viral vector. Upon viral establishment and systemic spreading into the upper parts of the plants, yellow mosaics occur as first symptoms on leaves. In the further course of plant development, the disease entails leaf necrosis and increased susceptibility to frost damage. Thanks to the *rym4* and *rym5* allelic variants of the *HvEIF4E* gene, more than two thirds of current European winter barley cultivars are resistant to BaYMV and BaMMV. However, several strains of BaYMV and BaMMV have already overcome *rym4-* and *rym5*-mediated resistance. Accordingly, new resistance-conferring alleles are needed for barley breeding. Therefore, we performed targeted mutagenesis of the *EIF4E* gene by Cas9 endonuclease in BaMMV/BaYMV-susceptible winter barley cv. “Igri”. Small insertions were generated, resulting in a shift of the translational reading frame, thereby causing the loss-of-function of *EIF4E*. The mutations occurred in the homozygous state already in the primary mutants. Their progeny proved invariably homozygous and fully resistant to mechanical inoculation with BaMMV. *EIF4E* knockout plants showed normal growth habit and produced grains, yet exhibited a yield penalty.

## Introduction

The Eukaryotic Translation Initiation Factor 4E (eIF4E) and its isoform [eIF(iso)4E] are known as susceptibility factors for potyvirus infection in a variety of plant species such as melon, tomato and pepper (Wang and Krishnaswamy, 2012). The eIF4E protein interacts with the 5′ 7-methylguanosine (m^7^G) cap of eukaryotic mRNA to recruit the translation complex for protein biosynthesis. Potyviruses are single stranded (+)RNA viruses that mimic the structure of the eukaryotic mRNA’s m^7^G cap by their genome-linked viral protein (VPg) cap, thus taking advantage of host metabolism for translation of their RNA (Sanfaçon, 2015). The plasmodiophorid *Polymyxa graminis* serves as a vector of *Barley yellow mosaic virus* (BaYMV) and *Barley mild mosaic virus* (BaMMV), both of which are bymoviruses of the Potyvirideae family. *P. graminis* transmits these viruses mainly to winter barley seedlings, particularly under cool and moist weather conditions in autumn, which results in high yield losses and renders bymoviruses major pathogens in Europe and Asia. Barley was among the first species in which resistance-mediating alleles of the *EIF4E* gene were described and utilized in breeding (Kanyuka et al., 2005; Stein et al., 2005). The *rym4* and *rym5* alleles of *HvEIF4E* carry mutations that result in amino acid changes in the binding domain of the encoded protein. Consequently, the interaction between the host’s eIF4E and the viral RNA cap-like structure formed by the genome-linked viral protein (VPg), is hampered, thereby preventing the translation of viral RNA and thus the replication and spread of the virus in the plant (Li and Shirako, 2015). In total, seven allelic variants of the *EIF4E* gene have been shown to confer resistance to different isolates of the BaMMV/BaYMV complex [reviewed in Jiang et al. (2020)]. Based upon this principle, barley cultivars resistant to BaYMV and BaMMV have been bred and widely grown in temperate climate regions. However, the comprehensively used *rym4*- and *rym5*-based resistances have already been overcome by some strains such as BaYMV-2 (overcoming *rym4*) as well as BaMMV-Sil and BaMMV-Teik (overcoming *rym5*) (Habekuss et al., 2008; Jiang et al., 2020). Consequently, there is an urgent need for new resistance-conferring *HvEIF4E* alleles or novel resistance mechanisms, especially in winter barley breeding. Besides the *EIF4E* alleles *rym4* and *rym5*, several other bymovirus resistance loci have been described in barley [reviewed in Jiang et al. (2020)]. Most of these are recessive and were found in Asian landraces. However, only for *rym1/rym11*, the responsible gene, i.e., the *Protein Disulfide Isomerase-Like 5-1* (*PDIL5-1*), has been identified (Yang et al., 2014). Nevertheless, winter barley breeding still relies on *rym4* and *rym5*, since the introduction of new resistance sources into current elite lines requires time-consuming backcrosses. Taking advantage of the CRISPR-associated (Cas) endonuclease technology (Koeppel et al., 2019), targeted knock-outs of *EIF4E* were reported to be associated with Potyvirus resistance in other plant species such as cucumber and cassava (Chandrasekaran et al., 2016; Gomez et al., 2019). In barley, however, only non-synonymous single nucleotide polymorphisms (SNPs) have been described that lead to amino acid changes in the mRNA cap-binding domain of eIF4E. Therefore, it is assumed that the loss-of-function of *EIF4E* might be lethal in barley even though *HvEIF*(*iso*)*4E* does exist as a conserved paralog (Yang et al., 2016). Here, we addressed four positions in the *EIF4E* gene previously described to carry different SNPs in the *rym4* and *rym5* resistance alleles of the virus-susceptible winter barley cv. “Igri” using Cas9 and accordingly customized guide RNAs (gRNAs). *Via Agrobacterium*-mediated DNA transfer to embryogenic pollen cultures, target motif-specific mutant plants were generated and their progeny were tested for resistance against BaMMV.

## Materials and Methods

### Target Selection

**FIGURE 1:**
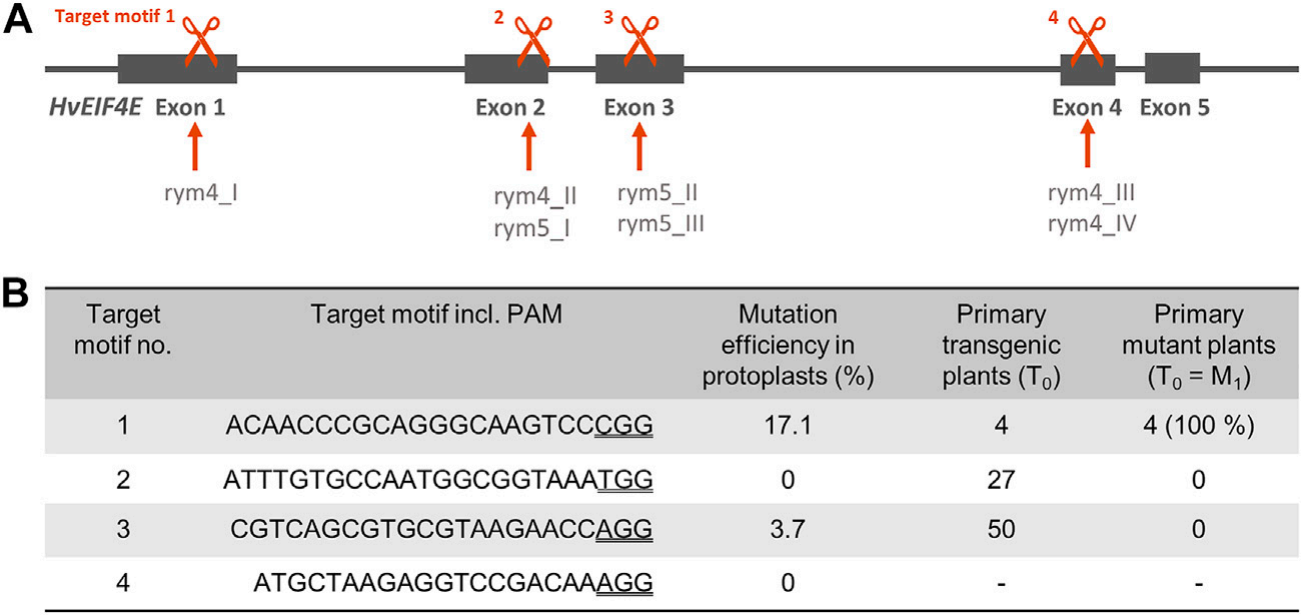
**(A)** Gene structure of *EIF4E*, positions of previously described single nucleotide polymorphisms in *rym4* and *rym5* resistance alleles and positions of Cas9/gRNA target motifs for site-directed mutagenesis in *Hordeum vulgare*. Targets indicated by scissors symbols, genomic *EIF4E* sequence shown by grey line and exons by grey boxes. **(B)** Summary of target sequences, results of protoplast-based pre-validation of mutation efficiency, number of regenerated plants upon *Agrobacterium*-mediated DNA transfer to embryogenic pollen cultures of winter barley cv. “Igri”, and mutant plants detected amongst transgenic regenerants.

As targets for Cas9-induced mutagenesis, regions within the *HvEIF4E* gene (Gene ID: 100527994) on Chromosome 3 were selected, in which previously non-synonymous single nucleotide polymorphisms (SNPs) have been described for the resistance-conferring *rym4* and *rym5* alleles which are, respectively, associated with four and three SNPs (see Figure 1A). The aim was to induce knockout mutations as well as new alleles by random nucleobase exchanges at the target sites. Within the four target regions, target motifs were selected based on available NGG protospacer-adjacent motifs (PAM). Target motifs including their PAMs are shown in Figure 1B. An off-target analysis was performed by comparison of the selected target motifs with the barley reference genome “Morex” Version 3 and the pan-genome sequence of “Igri” using the GrainGenes BLAST Service (Priyam et al., 2019). Only for target motif 3, a similar sequence is present on Chromosome 1 but a 2-bp mismatch within the seed region renders this sequence a rather unlikely off-target. For target motifs 1, 2, and 4, no potential off-targets were identified in the “Morex” genome v3 (see Suppl. S1).

### Vector Cloning

The target-specific sequences of the four gRNAs (see Figure 1B; Supplementary Table S1) were ordered as forward and reverse DNA oligo-nucleotides, hybridized and individually cloned in the format of double-stranded DNA into the plasmid pSH121 [GeneBank-ID: MW145140.1; Gerasimova et al. (2018)]. This generic vector contains the *Oryza sativa U3* (*OsU3*) promoter in front of a gRNA scaffold as well as the maize codon-optimized *cas9* gene under control of the *Zea mays Polyubiquitin 1 (ZmUBI1)* promoter. From each target-specific pSH121 derivative named pInt_EIF4E_TM1, pInt_EIF4E_TM2, pInt_EIF4E_TM3, and pInt_EIF4E_TM4, the *Sfi*I fragment including the gRNA and *cas9* expression units was transferred into the binary vector p6i-2x35S-TE9 (Gerasimova et al., 2020) that carries in its transfer-DNA an *hpt* gene under the control of a doubled-enhanced CaMV35S promoter for plant selection, resulting in the respective binary vectors pBin_EIF4E_TM1, pBin_EIF4E_TM2, pBin_EIF4E_TM3, and pBin_EIF4E_TM4.

### Pre-Validation of Constructs *via* Protoplast Transformation

The intermediate pSH121 derivatives were first tested by transient expression in mesophyll protoplasts isolated from leaves of one week-old, etiolated seedlings of winter barley cv. “Igri” based on the protocol of Shan et al. (2014). In brief, thirty leaves of barley seedlings were chopped into small fragments using a razor blade, and cell walls were digested with Macerozyme R-10 and cellulase R-10 (DUCHEFA BIOCHEMIE B.V, Haarlem/Netherlands). Protoplasts were purified by sieving, and after PEG-mediated transformation with pInt_EIF4E_TM1 to pInt_EIF4E_TM4, they were incubated for 60 h at 21°C in the dark. All protoplast transformations were conducted in three replications. To check for transformation efficiency, a *GFP* construct was used to transform a control sample of protoplasts. After incubation, the *GFP* expressing portion of protoplasts of this control sample was determined using an epifluorescence microscope (AX200M, Zeiss, Oberkochen/Germany), and DNA was extracted from the other protoplast samples transformed using the intermediate *cas9*/gRNA constructs pInt_EIF4E_TM1, pInt_EIF4E_TM2, pInt_EIF4E_TM3, and pInt_EIF4E_TM4. Around 150 bp of the target regions were amplified using specific primers (see Supplementary Table S1), followed by deep-sequencing of amplicons, which was performed by a commercial service provider on an Illumina MiSeq platform. Mutation efficiencies were calculated individually for each replicate as proportion of sequencing reads with mutation in relation to the total number of reads including those with the wild-type sequence.

### Agrobacterium-Mediated Transformation Using Embryogenic Barley Pollen

The binary vectors pBin_EIF4E_TM1, pBin_EIF4E_TM2, and pBin_EIF4E_TM3 were transfected by electroporation into the *Agrobacterium* strain LBA4404 harboring the hypervirulence-conferring plasmid pSB1 (Kumlehn et al., 2006). In brief, pre-mitotic (highly vacuolated) microspores were isolated from winter barley cv. “Igri”. After microspore cultivation for 1 week in KBP medium (macro and micro nutrients, L-glutamine and maltose) to initiate cell proliferation, resultant embryogenic pollen were subjected to DNA transfer by co-cultivation with *Agrobacterium* carrying the binary vectors as mentioned above in CK medium (macro and micro nutrients, maltose, acetosyringone, MES, and phosphate buffer). Then, transgenic plantlets were generated from embryogenic structures cultivated on KBP4PT (macro and micro nutrients, L-glutamine, maltose, hygromycin, Timentin, and Phytagel) followed by K4NBT medium (macro and micro nutrients, with increased dosage of CuSO_4_, L-glutamine, maltose, hygromycin, Timentin, and Phytagel) under hygromycin selection as previously described in detail by Kumlehn et al. (2006).

### Genotyping of Primary Transgenics and Mutants

Leaf samples were taken from primary transgenic plants and DNA was extracted and analyzed for the presence of the *cas9* transgene by PCR using specific primers (see Supplementary Table S1). The plants were screened for mutations by PCR amplification of the target regions using specific primers (see Supplementary Table S1) followed by Sanger sequencing of the amplicons.

### BaMMV Inoculation and Resistance Screening by DAS-ELISA

**FIGURE 2:**
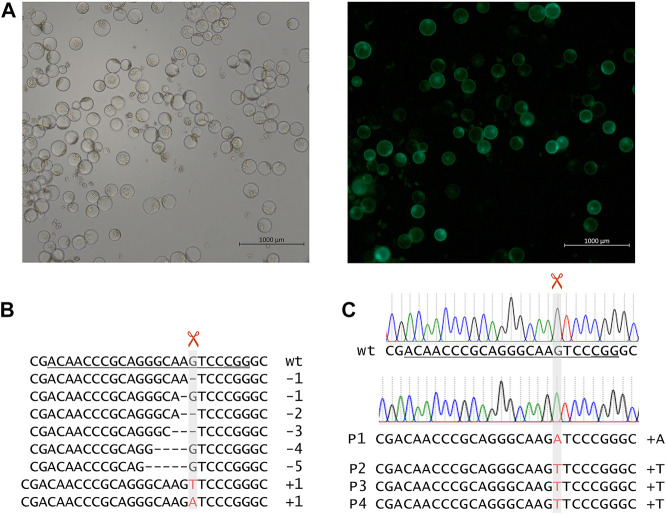
**(A)** Mesophyll protoplasts of winter barley cv. “Igri” after PEG-mediated transformation with a *GFP*-carrying vector, after 60 h of incubation at 21°C. These protoplasts serving as positive control for viability and genetic transformation, respectively, were recorded using bright field **(left)** and epifluorescence **(right)** microscopy. **(B)** Deep-sequencing of amplicons of target region 1 after transformation of barley protoplasts using vectors carrying *cas9* and target motif 1-specific gRNA expression units. **(C)** Chromatograms of Sanger sequencing target region 1 of wild-type “Igri” as compared with primary mutant plants. The unambiguous DNA sequence indicates homozygosity of the inserted A nucleotide. Grey vertical bars: cleavage positions, horizontal lines: deletions, red letters: insertions, wt: wild-type sequence, target motifs underlined with PAMs being double underlined, +A: 1-bp insertion of adenine nucleobase, +T: 1-bp insertion of thymine nucleobase.

To screen progeny of primary mutants P1, P3, and P4 for Bymovirus resistance, plants were mechanically inoculated with BaMMV according to a protocol of Habekuss et al. (2008) which had been established as a more reliable alternative to vector-mediated virus infection using the soil-borne plasmodiophorid *Polymyxa graminis*. Depending on the number of grains obtained from primary mutants, 13, 9, and 16 M2 siblings were grown and screened (see Figure 2C). For this purpose, a total of 38 seedlings were cultivated in a growth chamber at 12°C and 16 h photoperiod and inoculated twice with an interval of five to 7 days at the three-leaf stage with leaf sap of BaMMV-ASL-infected barley plants. Six to 8 weeks after the first inoculation, visible symptoms as shown in Figure 2B were assessed. Additionally, the plants were screened for virus particles by double-antibody sandwich enzyme-linked immunosorbent assay (DAS-ELISA). Plants exhibiting an extinction *E*_405_ of 0.1 or below were considered resistant to BaMMV. Simultaneously, DNA was extracted from leaf samples of all test plants, PCR was performed for the *cas9* transgene and the target region was amplified and sequenced as described above. As control, non-transformed (non-edited) “Igri’ plants, referred to as “wild-type”, were included to the tests.

### Yield Data

After resistance screening, M2 plants were cultivated in a glasshouse with 18–20°C/ 12–14°C day/night with 16 h light to maturity, harvested and ears were threshed. The total grain number of each individual plant was counted and total grain weight was measured. Thousand grain weight (TGW) was calculated as

$$TGW=\frac{Grain\;weight}{Grain\;number}*1000$$

An analysis of variance (ANOVA) and Post-Hoc Test (Tukey HSD) were performed using the statistics software R version 3.6.1 (R Core Team, 2019). Plots were generated with R package ggplot2 (Wickham, 2016).

## Results

**FIGURE 3:**
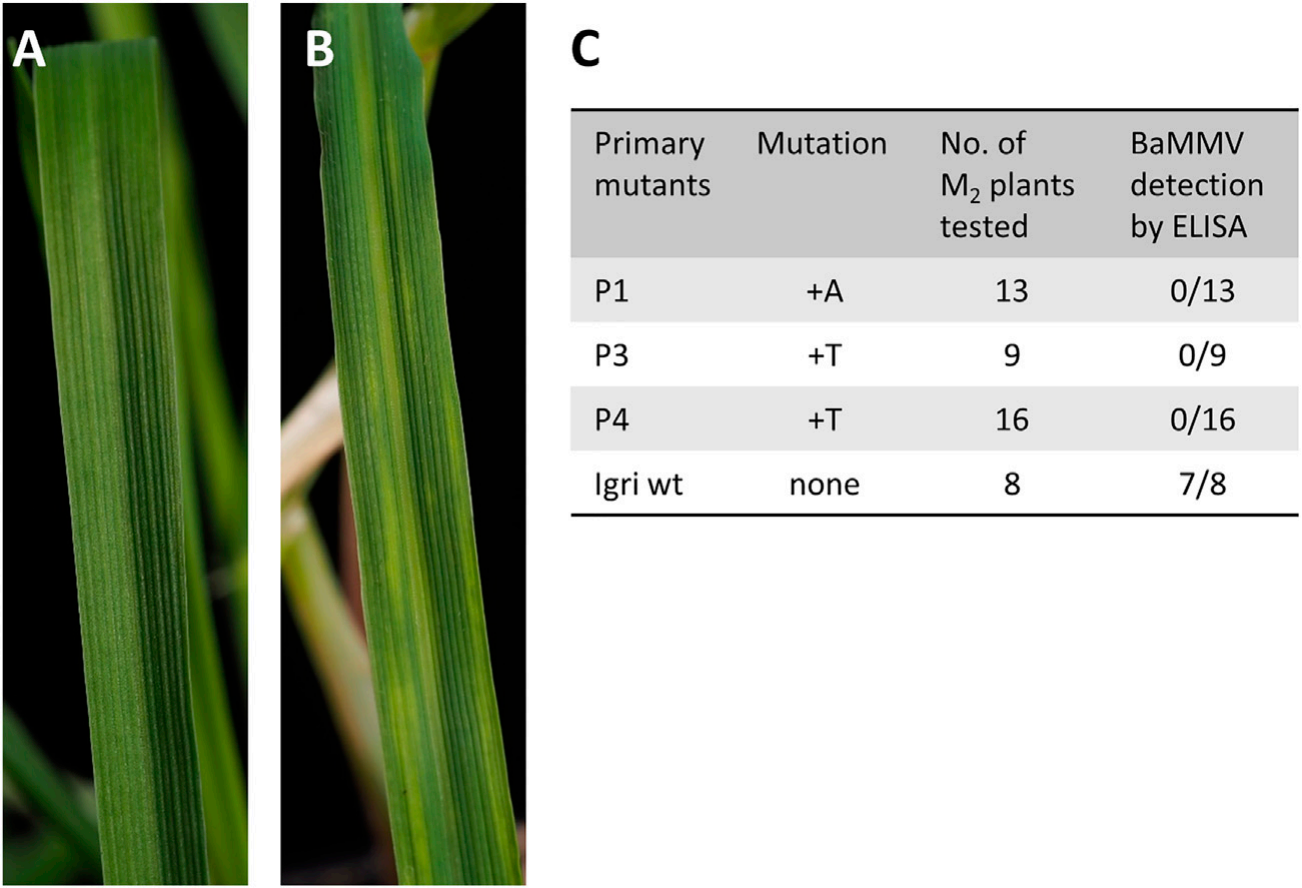
**(A)** Healthy leaf of winter barley “Igri” approximately 2 months after sowing. **(B)** Symptoms *of Barley Mild Mosaic Virus* (BaMMV) infection 7 weeks after mechanical inoculation of wild-type “Igri”. **(C)** Results from mechanical BaMMV inoculation of M2 plants followed by DAS-ELISA for detection of virus particles and concomitant molecular characterization of mutation status. All M2 plants carried the same mutation as their parental plant, confirming homozygosity of the latter. All mutants proved resistant against BaMMV infection.

After cloning pInt_EIF4E_TM1, pInt_EIF4E_TM2, pInt_EIF4E_TM3, and pInt_EIF4E_TM4 bearing *cas9* and target motif-specified gRNA expression units, a barley protoplast-based test assay was performed to pre-validate the construct performance in terms of mutagenesis efficiency. After transformation and incubation of protoplasts (see Figure 3A), their genomic DNA was extracted and PCR amplicons of target regions were deep-sequenced. For each target motif, several hundred reads were obtained. The alignment of the sequencing reads to the “Morex” reference sequence revealed that the proportions of mutated sequence reads related to the total read numbers (including wild-type) were as high as 17.1%, with the best results being achieved in target motif 1 (see Figure 1B). The most frequent mutations observed in protoplasts were small deletions of 1–5 base pairs or insertions of one base pair (A or T) at the expected Cas9 cleavage site residing three to four base pairs upstream of the protospacer-adjacent motif (PAM) (Jinek et al., 2012) as shown in Figure 3B. Unfortunately, only after sequencing, the PAM of target motif 4 was found to be unsuitable, because it features a single nucleotide polymorphism in “Igri” as compared to the barley genomic reference sequences of cv. “Morex”.

After cloning the binary vectors pBin_EIF4E_TM1, pBin_EIF4E_TM2, and pBin_EIF4E_TM3 with the expression cassettes for *cas9* and the gRNAs against target motifs 1, 2, and 3, *Agrobacterium*-mediated DNA transfer to embryogenic pollen cultures of winter barley cv. “Igri” was used to produce transgenic plants. Addressing target motifs 1, 2, and 3, four, twenty-seven and fifty primary transgenic plants were obtained from one, five and one transformation experiments, respectively. All regenerated plants were PCR-positive for the *cas9* gene and were subjected to Sanger sequencing of the addressed target motifs. Whereas independent 1-bp insertions of T or A were found three base pairs upstream of the PAM in each of the four plants carrying pBin_EIF4E_TM1 (see Figure 3C), no mutations whatsoever were detected in plants transformed using pBin_EIF4E_TM2 and pBin_EIF4E_TM3. The undisturbed chromatograms obtained for the four primary mutant plants were taken as indication for homozygosity of the induced mutations. Progenies of three independent primary mutants were genotyped by PCR screening for the *cas9* transgene and by Sanger sequencing of the target region. All individuals carried the same mutations known from their respective parent, thereby confirming its homozygous state. In addition, these M2 plants were mechanically inoculated with BaMMV, whereby all of them proved resistant to the infection (see Figure 2C).

The insertion of one base pair at position 169 of the *EIF4E* coding sequence results in a shift of the reading frame during translation of the mRNA, which itself causes a non-sense amino acid sequence downstream of this insertion site including a premature stop codon at position 306 (after approx. one third) of the coding sequence (see Supplementary Material).

**FIGURE 4:**
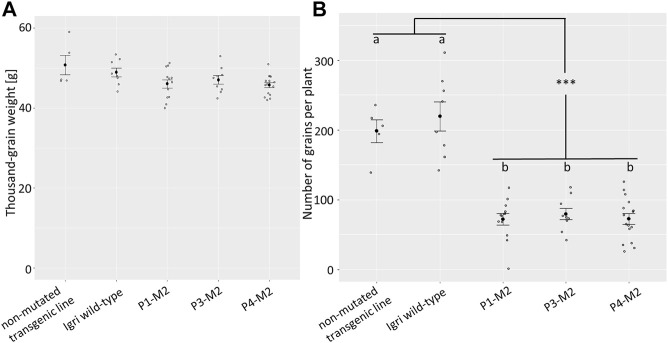
**(A)** Thousand-grain weight of three M2 populations (9–16 siblings each), compared to non-mutated segregants from a comparable transformation experiment (*n* = 5) and “Igri” wild-type (*n* = 8), grown under the same conditions. Black dots show the average weights of 1,000 grains in g, error bars indicate standard errors. Statistically significant differences were not detectable (Tukey’s test, *p* ≥ 0.05). **(B)** Total number of grains per plant of three M2 populations (9–16 siblings each), compared to non-mutated segregants from a comparable transformation experiment (*n* = 5) and “Igri” wild-type (*n* = 8), grown under the same conditions. Black dots show the average numbers of grains per plant, error bars indicate standard errors. Samples without statistically significant differences (Tukey’s test, *p* > 0.8) are indicated by same letters, statistically significant differences between the two groups indicated by ‘***’ (Tukey’s test, *p* < 0.0001).

Yield was assessed based on thousand-grain weight and grain number per plant (GNP) of the three analyzed M2 families. The thousand-grain weight of the mutant families P1 (mean 46 g, ±3.5), P3 (47 g ± 3.1), and P4 (45.7 g, ±2.4) was on a par with that of non-mutated, transgenic segregants from a similar experiment (50.7 g, ±4.9) and of “Igri” wild-type (48.9 g, ±3.2) grown under the same glasshouse conditions (see Figure 4A; Supplementary Table S2). According to a one-way analysis of variance (ANOVA) followed by Tukey’s post-hoc test, no significant differences in the thousand-grain weight were observed (*p* ≥ 0.05). However, the total grain yield per plant was decreased owing to a reduction in the number of grains produced per plant. This phenomenon was consistent across all three analyzed mutant lines (P1: 72 grains, ±29; P3: 79 grains, ±25; P4: 72 grains, ±30) which were compared to wild-type (220 grains, ±59) and non-mutated segregants (198, ±37) (*p* < 0.001; see Figure 4B; Supplementary Table S2).

## Discussion

The pre-validation of *cas9*/gRNA constructs *via* protoplast assay proved conclusive for targeted mutagenesis at the whole-plant level. Notably, comparatively low mutation rates were expected, because targeting specifically those regions known for resistance-conferring mutations required trade-offs with respect to some efficiency-related criteria for the selection of target motifs (Kumlehn et al., 2018). Target motifs 2 and 3 showed zero to very low mutation rates in protoplasts and so they did in *cas9*/gRNA transgenic plants. Positional effects might have played a role, since both target motifs mutated with low efficiencies are located close to each other on exons 2 and 3. Furthermore, the protospacer-adjacent motif (PAM) of target motif 4 was found to be unsuitable only after sequencing, because it features a single nucleotide polymorphism in “Igri” as compared to the barley genomic reference sequences that were used for target motif selection (see also Supplementary Material). For target motif 1, the insertions found in transgenic plants were also amongst the most frequent mutations detected in the respective protoplast assay (see Figure 3B). The mutations proved instantly homozygous in all four primary transgenic plants, most likely due to the particularity of the applied transformation method which involves gene transfer to haploid cells followed by whole-genome duplication (Kumlehn et al., 2006; see Figures 2C, 3C). To the best of our knowledge, this represents the first example of generating homozygous mutations via transfer of *cas9*/gRNA-encoding DNA to haploid cells that are capable of giving rise to (doubled haploid) plants. This enables us to evaluate phenotypes of homozygous mutants already in early generations after transformation. As a trade-off, these plants are most likely also homozygous for the *cas9*/gRNA-transgene. However, this disadvantage is considered irrelevant in the scientific exemplification demonstrated in the present study, in which also a rather outdated barley cultivar was used to take advantage of its amenability to genetic transformation. Moreover, there is the option of crossing these mutants with wild-type plants, followed by selfing of F1 plants, which would readily result in T-DNA-free segregants that still carry the mutated eif4e allele in the homozygous state. Also, the mutation at the target motif itself prevents the occurrence of new mutation events by disturbing the gRNA to bind to the target sequence. To exclude any effects of the transgene itself, five non-mutated plants carrying events of the same transgene except from the 20 bp target-specific gRNA sequence were used as control and their grain yield was on a par with that of “Igri” wild-type plants.

The one-base pair insertion between position 169 and 170 in the *EIF4E* coding sequence (position 400-401 in the genomic sequence) causes a frameshift in the translational reading frame, resulting not only in a nonsense amino acid sequence downstream of the mutation, but also in a premature stop codon at position 265 bp of the coding sequence (see Supplementary Material). Consequently, the resultant protein sequence is non-functional with very high certainty. Owing to the loss-of-function of the eIF4E protein, the plant lacks one of its most important interaction partners for bymoviruses (Wang and Krishnaswamy, 2012). That mechanism has been reported to confer broad resistance to several bymoviruses in a number of dicotyledonous plants as, for instance, cucumber, tomato and *Arabidopsis* (Chandrasekaran et al., 2016; Pyott et al., 2016; Atarashi et al., 2020; Moury et al., 2020). However, in a temperate cereal such as barley, the full knock-out of *EIF4E* was rather expected to be lethal; Yang et al. (2016) screened over 2,900 wild and domesticated barley accessions and identified 65 haplotypes for *HvEIF4E*, of which 19 were associated with resistance to bymoviruses. The allelic diversity included various non-synonymous point mutations, whereas knockout alleles were not present. Hence, it was concluded that a knockout of *HvEIF4E* cannot be complemented by its paralogue *HvEIF*(*iso*)*4E* without disadvantage for the plants. Hofinger et al. (2011) described the haplotype diversity of *EIF4E* in barley in comparison to its paralogue *EIF*(*iso*)*4E*. While a rather high diversity was described for the first, most likely due to positive selection pressure by co-evolution with bymoviruses, rather low haplotype diversity was found for the latter, suggesting different functions of *EIF4E* and *EIF(iso)4E* in barley. Nevertheless, during our experiments in climate chamber and glasshouse, *HvEIF4E* knockout plants grew vigorously and produced grains. However, the total number of grains per plant was significantly affected. Previous publications on targeted *EIF4E* knockouts with resulting resistance to Potyviruses in other plant species unfortunately did not contain yield data to refer to here (Chandrasekaran et al., 2016; Gomez et al., 2019). Since isogenic wild-type lines for barley accessions with *rym4*- or *rym5*-mediated resistance are not yet available, it is not even known whether these widely used resistances are associated with yield reduction. The results of our study need confirmation under field conditions to elucidate whether the *eif4e* knockout represents a viable novel source of resistance to bymoviruses in barley, which most likely, in contrast to *rym4* and *rym5* and the other alleles known at this locus, may be effective to all strains of BaMMV and BaYMV. In the event that the yield penalty seen in the present investigation will be confirmed under field conditions, the option remains for future biotechnological approaches to generate mutant alleles with sufficient functionality for the plant by inducing in-frame mutations, such as via base editing, so that a recruitment of the corresponding gene products by bymoviruses is no longer possible.

The availability of *eif4e* knockout mutants further offers the opportunity to dissect the effects of the individual SNPs jointly present in the *rym4*- and *rym5*-alleles as well as to elucidate the impact of various *EIF4E* alleles on yield, which could be achieved via complementation using synthetic alleles each carrying only one of those SNPs or various combinations thereof. The identification of effective SNPs will facilitate further efforts to generate novel resistance-conferring alleles by precise genome editing approaches.

## Data Availability

The original contributions presented in the study are included in the article/Supplementary Material, further inquiries can be directed to the corresponding author.
