# Supplementary material for: Targeted Knockout of Eukaryotic Translation Initiation Factor 4E Confers Bymovirus Resistance in Winter Barley
Source: Front Genome Ed. 2021 Nov 29;3:784233. doi: 10.3389/fgeed.2021.784233 (PMC8667817; doi:10.3389/fgeed.2021.784233)
Supplement: Supplementary file 3 [file DataSheet1.DOCX]

**Off-target analysis:**

Target Motif 1

5’-TGGTTCGACAACCCGCAGGGCAAGTCCCGGGCGGT-3’

>Query_1:1-35_alignment_gnl|Hv-Morex3|chr3H:612458327-612458361

TGGTTCGACAACCCGCAGGGCAAGTCCCGGGCGGT

|||||||||||||||||||||||||||||||||||

TGGTTCGACAACCCGCAGGGCAAGTCCCGGGCGGT

>Query_1:1-35_alignment_gnl|HvIgri|chr3H:617019814-617019848

TGGTTCGACAACCCGCAGGGCAAGTCCCGGGCGGT

|||||||||||||||||||||||||||||||||||

TGGTTCGACAACCCGCAGGGCAAGTCCCGGGCGGT

Target Motif 2

5’-ACCCCATTTGTGCCAATGGCGGTAAATGGACCATC-3’

>Query_1:1-35_alignment_gnl|Hv-Morex3|chr3H:612460127-612460161

ACCCCATTTGTGCCAATGGCGGTAAATGGACCATC

|||||||||||||||||||||||||||||||||||

ACCCCATTTGTGCCAATGGCGGTAAATGGACCATC

>Query_1:1-35_alignment_gnl|HvIgri|chr3H:617021506-617021540

ACCCCATTTGTGCCAATGGCGGTAAATGGACCATC

|||||||||||||||||||||||||||||||||||

ACCCCATTTGTGCCAATGGCGGTAAATGGACCATC

>Query_1:1-35_alignment_gnl|Hv-Morex3|chr1H:244446841-244446875

ACCCCATTTGTGCCAATGGCGGTAAATGGACCATC

||||||||||||||||||| ||||||||||||||

ACCCCATTTGTGCCAATGGTCGTAAATGGACCATC

>Query_1:1-35_alignment_gnl|HvIgri|chr1H:245488239-245488273

ACCCCATTTGTGCCAATGGCGGTAAATGGACCATC

||||||||||||||||||| ||||||||||||||

ACCCCATTTGTGCCAATGGTCGTAAATGGACCATC

Target Motif 3

5’-GGAGCAGTCGTCAGCGTGCGTAAGAACCAGGAAAG-3’

>Query_1:1-35_alignment_gnl|Hv-Morex3|chr3H:612460340-612460374

GGAGCAGTCGTCAGCGTGCGTAAGAACCAGGAAAG

|||||||||||||||||||||||||||||||||||

GGAGCAGTCGTCAGCGTGCGTAAGAACCAGGAAAG

>Query_1:1-35_alignment_gnl|HvIgri|chr3H:617021719-617021753

GGAGCAGTCGTCAGCGTGCGTAAGAACCAGGAAAG

|||||||||||||||||||||||||||||||||||

GGAGCAGTCGTCAGCGTGCGTAAGAACCAGGAAAG

Target Motif 4

5’-CAGGAGGATGCTAAGAGGTCCGACAAAGGCGCCAA-3’

>Query_1:1-35_alignment_gnl|Hv-Morex3|chr3H:612462700-612462734

CAGGAGGATGCTAAGAGGTCCGACAAAGGCGCCAA

|||||||||||||||||||||||||||||||||||

CAGGAGGATGCTAAGAGGTCCGACAAAGGCGCCAA

>Query_1:1-35_alignment_gnl|HvIgri|chr3H:617022876-617022910

CAGGAGGATGCTAAGAGGTCCGACAAAGGCGCCAA

|||||||||||||||||||||||||||| ||||||

CAGGAGGATGCTAAGAGGTCCGACAAAGCCGCCAA

*EIF4E*_wild-type_cds

MAEDTETRPASAGAEEREEGEIA

ATGGCGGAGGACACGGAGACGAGGCCCGCGTCGGCGGGCGCGGAGGAGAGGGAGGAGGGGGAGATCGCG

DDGDGSAAAAAGRVSAHPLENAW

GACGACGGAGACGGGTCCGCGGCGGCGGCGGCCGGGCGCGTCAGCGCCCACCCTCTGGAGAACGCCTGG

Target motif 1

TFWFDNPQGKSRAVAWGSTIHPI

ACCTTCTGGTTCGACAACCCGCAGGGCAAGTCCCGGGCGGTGGCCTGGGGGAGCACCATCCACCCCATC

HTFSTVEDFWSLYNNIHHPSKLN

CACACCTTCTCCACCGTCGAGGACTTCTGGAGCCTTTACAACAATATTCATCACCCTAGCAAGTTGAAT

Target mo-

VGADFHCFKDKIEPKWEDPICAN

GTTGGAGCCGACTTCCATTGCTTCAAGGATAAGATTGAGCCAAAATGGGAAGACCCCATTTGTGCCAAT

tif 2

GGKWTISCGKGKSDTFWLHTLLA

GGCGGTAAATGGACCATCAGTTGTGGCAAAGGGAAATCTGACACATTTTGGTTGCATACTTTGCTGGCA

Target motif 3

LIGEQFDFGDEICGAVVSVRKNQ

TTGATTGGTGAACAATTCGACTTTGGTGATGAAATTTGCGGAGCAGTCGTCAGCGTGCGTAAGAACCAG

ERVAIWTKNAANETAQISIGKQW

GAAAGAGTAGCTATCTGGACTAAAAATGCTGCCAATGAAACTGCTCAGATAAGCATCGGTAAGCAGTGG

Target motif 4

KEFLDYKDSIGFVVHEDAKRSDK

AAGGAGTTTCTGGACTACAAGGACTCCATTGGATTCGTCGTTCATGAGGATGCTAAGAGGTCCGACAAA

GAKNRYTV*

GGCGCCAAGAACCGCTACACGGTTTGA

*eif4e*_A169_cds

MAEDTETRPASAGAEEREEGEIA

ATGGCGGAGGACACGGAGACGAGGCCCGCGTCGGCGGGCGCGGAGGAGAGGGAGGAGGGGGAGATCGCG

DDGDGSAAAAAGRVSAHPLENAW

GACGACGGAGACGGGTCCGCGGCGGCGGCGGCCGGGCGCGTCAGCGCCCACCCTCTGGAGAACGCCTGG

TFWFDNPQGKIPGGGLGEHHPPH

ACCTTCTGGTTCGACAACCCGCAGGGCAAGATCCCGGGCGGTGGCCTGGGGGAGCACCATCCACCCCAT

PHLLHRRGLLEPLQQYSSP*QVE

CCACACCTTCTCCACCGTCGAGGACTTCTGGAGCCTTTACAACAATATTCATCACCCTAGCAAGTTGAA

CWSRLPLLQG*D*AKMGRPHLCQ

TGTTGGAGCCGACTTCCATTGCTTCAAGGATAAGATTGAGCCAAAATGGGAAGACCCCATTTGTGCCAA

WR*MDHQLWQREI*HILVAYFAG

TGGCGGTAAATGGACCATCAGTTGTGGCAAAGGGAAATCTGACACATTTTGGTTGCATACTTTGCTGGC

IDW*TIRLW**NLRSSRQRA*EP

ATTGATTGGTGAACAATTCGACTTTGGTGATGAAATTTGCGGAGCAGTCGTCAGCGTGCGTAAGAACCA

GKSSYLD*KCCQ*NCSDKHR*AV

GGAAAGAGTAGCTATCTGGACTAAAAATGCTGCCAATGAAACTGCTCAGATAAGCATCGGTAAGCAGTG

EGVSGLQGLHWIRRS*GC*EVRQ

GAAGGAGTTTCTGGACTACAAGGACTCCATTGGATTCGTCGTTCATGAGGATGCTAAGAGGTCCGACAA

RRQEPLHGL

AGGCGCCAAGAACCGCTACACGGTTTGA

*eif4e*_T169_cds

MAEDTETRPASAGAEEREEGEIA

ATGGCGGAGGACACGGAGACGAGGCCCGCGTCGGCGGGCGCGGAGGAGAGGGAGGAGGGGGAGATCGCG

DDGDGSAAAAAGRVSAHPLENAW

GACGACGGAGACGGGTCCGCGGCGGCGGCGGCCGGGCGCGTCAGCGCCCACCCTCTGGAGAACGCCTGG

TFWFDNPQGKFPGGGLGEHHPPH

ACCTTCTGGTTCGACAACCCGCAGGGCAAGTTCCCGGGCGGTGGCCTGGGGGAGCACCATCCACCCCAT

PHLLHRRGLLEPLQQYSSP*QVE

CCACACCTTCTCCACCGTCGAGGACTTCTGGAGCCTTTACAACAATATTCATCACCCTAGCAAGTTGAA

CWSRLPLLQG*D*AKMGRPHLCQ

TGTTGGAGCCGACTTCCATTGCTTCAAGGATAAGATTGAGCCAAAATGGGAAGACCCCATTTGTGCCAA

WR*MDHQLWQREI*HILVAYFAG

TGGCGGTAAATGGACCATCAGTTGTGGCAAAGGGAAATCTGACACATTTTGGTTGCATACTTTGCTGGC

IDW*TIRLW**NLRSSRQRA*EP

ATTGATTGGTGAACAATTCGACTTTGGTGATGAAATTTGCGGAGCAGTCGTCAGCGTGCGTAAGAACCA

GKSSYLD*KCCQ*NCSDKHR*AV

GGAAAGAGTAGCTATCTGGACTAAAAATGCTGCCAATGAAACTGCTCAGATAAGCATCGGTAAGCAGTG

EGVSGLQGLHWIRRS*GC*EVRQ

GAAGGAGTTTCTGGACTACAAGGACTCCATTGGATTCGTCGTTCATGAGGATGCTAAGAGGTCCGACAA

RRQEPLHGL

AGGCGCCAAGAACCGCTACACGGTTTGA
